# Scalp haircuts, keloids and blood-borne virus transmission risk in South Africa—The SHAKA study

**DOI:** 10.1371/journal.pone.0336213

**Published:** 2025-11-19

**Authors:** Nonhlanhla Khumalo, Avumile Mankahla, Stephen Korsman, Freedom Gumedze, Wisdom Basera, Anthia Ndyenga, Lwazi Mhlanti, Mashiko Setshedi, Ruanne Barnabas, C. Wendy Spearman, Mark W. Sonderup

**Affiliations:** 1 Hair and Skin Research Laboratory, Division of Dermatology, Groote Schuur Hospital, Faculty of Health Sciences University of Cape Town, Cape Town, South Africa; 2 Division of Dermatology, Department of Medicine and Pharmacology, Nelson Mandela Academic Hospital and Walter Sisulu University, Mthatha, Eastern Cape, South Africa; 3 Division of Medical Virology, NHLS and Groote Schuur Hospital and University of Cape Town, Cape Town, South Africa; 4 Division of Statistics, Department of Science, University of Cape Town, Cape Town, South Africa; 5 Burden of Disease Research Unit, South African Medical Research Council, Cape Town, South Africa; 6 Department of Medicine, Groote Schuur Hospital, Faculty of Health Sciences University of Cape Town, Cape Town, South Africa; 7 Division of Infectious Diseases, Department of Medicine, Massachusetts General Hospital, Harvard Medical School, Boston, Massachusetts, United States of America; 8 Division of Hepatology, Department of Medicine, Faculty of Health Sciences, University of Cape Town and Groote Schuur Hospital, Cape Town, South Africa; University of the Witwatersrand, SOUTH AFRICA

## Abstract

**Background:**

The most frequent transmission modes of blood-borne infections (BBI), including HIV, hepatitis B virus (HBV) and C (HCV), are well documented. South Africa, an HIV epicentre, with HBV endemicity and an additional HCV burden, raises the possibility of novel transmission means. A unique style of close shave haircut that can elicit *folliculitis keloidalis nuchea* (FKN), may produce bleeding during haircuts and possible BBI transmission. We designed a prospective case control study to evaluate the potential risk of BBI transmission.

**Methods:**

Men with FKN and non-FKN controls were recruited from 2 centres in South Africa, Cape Town and Mthatha. The presence of FKN was diagnosed by a dermatologist and clinical photographs independently corroborated by 3 other dermatologists not involved in the study. A comprehensive confidential questionnaire was administered to each participant interrogating risk factors for potential HIV, HBV, and HCV infection. Each participant was screened for HIV 1/2 (Alere Determine), HBsAg (Alere Determine) and HCV antibody (SD Bioline) using point of care tests. Positive HIV screens were confirmed with a second test, Vikia HIV 1/2 test (bioMerieux). Logistic regression analysis adjusting for the relevant confounder was used to assess the associations.

**Results:**

A total of 1163 men, median age 33.4 years [IQR 27.8–42.0], were evaluated. Those who screened positive for any viral infections were significantly older than those who did not, 37.1 [IQR 31.0–44.4) vs. 32.3 [IQR 26.9–41.0] years, p < 0.001, respectively. Overall, the seroprevalence of HIV, HBV, and HCV, was 17.2%, 6.9% and 0.4%, respectively. There was no significant difference in seroprevalence for these potential BBIs between those with FKN and without, p = 0.33, 0.66 and 0.29, respectively. For HIV co-infection with HBV and HCV, findings were similar, p = 0.64 and 0.51, respectively. When controlled for whether participants knowingly bleed during haircuts, HIV seropositivity was significantly more likely in those who regularly bleed, OR = 2.51 [95% CI 1.16–5.42], p = 0.02. Other transmission risk factors for HIV, were also significantly more likely – reported sexually transmitted genital lesions or discharge, aOR = 1.58 [95% CI 1.13–2.22], p = 0.01; and a tattoo/piercing informally done, aOR = 1.84, [95% CI 1.13–2.22], p = 0.01. Similarly, although overall HCV seroprevalence was low, those HIV/HCV co-infection was more likely in those who usually bleed with a haircut, aOR = 2.43; [95% CI 1.13–5.25]; p = 0.02, those with a sexually transmitted genital lesion or discharge, aOR = 1.58 [95% CI 1.13–2.21]; p = 0.01 and those with a tattoo/piercing informally done, aOR = 1.85 [95% CI 1.27–2.72]; p < 0.01. This was not so for HBV mono-infection, p = 0.89. Overall rates of known and recorded HBV vaccination in childhood were low, however in those with no viral infection, the rate of known vaccination was higher, 25.0% vs. 16.2%; p = 0.01 respectively.

**Conclusion:**

Haircut related bleeding but not FKN was associated with a higher prevalence of HIV and HIV/HCV co-infection. HBV risk was not increased and related to either vaccination and/or dominant early childhood acquisition risk of HBV in sub-Saharan Africa. HIV and HBV prevalence remains concerningly high. Risk reduction through public education is key to prevention.

## Introduction

Sub-Saharan Africa, with almost 70% of the global burden, is the epicentre of the global HIV/AIDS pandemic [1]. Concomitantly, hepatitis B virus (HBV) is endemic in the region. Of the estimated global 258 million people chronically infected with hepatitis B, 64.8 (95% UI 52.8–80.8) million people reside in Africa, where overall HBsAg positive seroprevalence is 5.4% (95% UI 4.4–6.8%) [2]. Approximately 7.8% (2.6 million) of those HIV positive in sub-Saharan Africa are HIV–HBV co-infected [3]. An estimated 8 million (95% UI 6–15 million) are infected with hepatitis C virus (HCV) in the WHO Africa region, while 6% of people living with HIV (PLWHIV) are HCV co-infected, with rates varying from 4.5% in southern to nearly 7% in western sub-Saharan Africa [4,5]. While regionally, predominant modes of transmission may vary, HIV, HBV and HCV are all blood- borne viruses.

South Africa shoulders a significant burden with 12.7% of adults (7.8 million) living with HIV, 2.9 million chronically infected with HBV and 400 000 with chronic HCV infection [6,7]. In South Africa, as for Africa, horizontal heterosexual transmission remains the dominant mode of HIV transmission [8]. In contrast, hepatitis B transmission occurs mostly horizontally between children before age 5 or perinatally from mother to child [9]. Risk of HBV chronicity ranges from 90% in perinatal infection to 25–60% risk between 6 months and 5 years of age. Hence chronic HBV infection is established long before adolescence and adulthood where HIV acquisition risk rises [9]. Globally, male to female ratios of chronic hepatitis B demonstrates a male preponderance of 2.6: 1 [10]. In South Africa, most of those with chronic HBV infection are HBeAg negative with typically lower HBV viral loads, however data from South Africa suggests that HIV has influenced this with HBV viral loads higher in those co-infected and up to 20% HBeAg positive [11]. These higher HBV viral loads enhance transmission risk as a blood-borne infection. Hepatitis C has a bimodal age distribution in South Africa, with younger age invariably associated with injecting drug use or sexual transmission in men who have sex with men [12].

As blood-borne infections, HBV, HCV, and HIV transmission can occur through percutaneous or mucosal contact with infectious blood or body fluids. Percutaneous modes of transmission besides needle stick injuries, include tattoos, body piercing and contaminated razors [13]. “Haircut associated bleeding” from a popular clean-shave haircut (colloquially called a “*chiskop”*) is performed using barber clippers, however the plastic comb-like attachment is removed, and the metal portion of the clipper blade applied directly onto the scalp against the grain of the hair. Additionally, shaving the head with a razor blade may also occur at the time of the clipper based close shave haircut. In Cape Town, the style is popular in more than 60% of African men [14]. Notably, the style of haircutting carries an elevated risk of ‘haircut associated bleeding” [15,16]. Micro-bleeding, not visually apparent, has been observed in 37% of clean-shave haircut users, confirmed on genetic testing for the blood specific RNA-marker, haemoglobin beta (HHB) in scalp swabs [17]. In a recent study, blood was detected in 42% and hepatitis B (HBV) in 8% of barber hair clippers, respectively [18]. Infection risk with informal barbers has been described elsewhere in sub-Saharan Africa [19]. Preventing blood borne infections in these novel settings, is challenging given the informal nature of the settings in which they occur with no clear public health policy governing such sectors. *Folliculitis keloidalis nuchea* (FKN) affects 10.5% of adult African males [14]. It is a primary scarring alopecia, although it is suggested that it may be due to the in-growing hair with a foreign body reaction to hair keratin retention within the dermis, such as pseudo-folliculitis barbae (beard shaving bumps). Characteristic and permanent scalp pimples and keloids are prone to injury and bleeding during these haircuts [18]. Haircuts often occur at informal barbershops with sub-optimal sterilization of razors and clippers. The popularity of this style of clean-shave haircut, poor sterilization facilities at most informal barbers and the frequency of haircut associated bleeding, we postulate that it raises concern for the potential transmission of blood-borne infections such as HIV, HBV, and HCV. Few prospective data exist on the potential risk of blood-borne viral transmission with clean-shave haircuts.

The primary aim was to compare the prevalence of HIV, HBV, HCV as well as HIV/HBV and HIV/HCV co-infection in patients with the clean-shave haircut who have FKN compared to controls with healthy scalps. Furthermore, using a confidential structured questionnaire, to potentially determine if the clean-shave hairstyle is a risk factor for HIV, HBV, HCV or HIV co-infection acquisition, in addition to potential factors such as age or potential confounding variables that may also increase risk of HIV, HBV or HCV acquisition.

## Methods

### Study design and groups

We performed a prospective case-control study with each case matched with 4 controls. Males with FKN and controls with healthy scalps were recruited from dermatology clinics. Controls were age-matched with mild localized non-scalp skin disease (e.g., acne, contact dermatitis, nummular eczema). Patients were recruited from 2 sites – Groote Schuur Hospital and its affiliated referral clinics in Cape Town, the Western Cape Province and the Nelson Mandela Hospital in Mthatha, the Eastern Cape Province. This allowed for heterogeneity and diversity to be representative of an urban, peri-urban and rural spectrum of participants.

Inclusion criteria were males older than 20 years, able to provide written consent and with either a clinical diagnosis of FKN or controls with healthy scalps, willing and able to consent and return for post-test counselling. Exclusion criteria were females, males <20 years old; other scalp disease or those refusing consent.

### Establishing the diagnosis of FKN

FKN at visit 1 was diagnosed by a Dermatologist or Dermatology specialist in training (with ≥2 years in training). Each participant’s scalp was photographed in a standardized manner (2 pictures, black background, camera on macro setting, automatic flash photography at 2 distances from the head of 50 cm and 30 cm for the 1^st^ and 2^nd^ picture, respectively). Three dermatologists not involved in the study graded the photographs of each participant from 0 (normal) to 3, denoting increasing severity of FKN. Agreement in 2/3 or 3/3 was used to allocate severity of FKN.

### Viral hepatitis and HIV testing

A comprehensive confidential questionnaire was completed by the recruiter interrogating risk factors for potential HIV, HBV and HCV infection. Pre-test counselling for HIV was performed. Blood was drawn. Recruitment commenced on 10/08/2016 with last participant recruitment on 23/11/2020.

Blood samples were appropriately transferred to the main study site in Cape Town. Sample processing and testing was as follows:

HBV: HBsAg tested using the Alere Determine HBsAg rapid test (98.6% sensitivity, 99.6% specificity; Alere Medical, Chiba, Japan). Concomitant formal laboratory HBsAg testing was performed, including indeterminate results using the ARCHITECT II system (*Abbott* Diagnostics, USA) No HBV DNA quantification was performed.

HCV: HCV antibody was evaluated using the SD BioLine HCV (Multi) rapid test (99.3% sensitivity, 100% specificity; Standard Diagnostics, Korea). Indeterminate results were re-processed in the central laboratory. Positive tests were further tested for the presence of HCV RNA to confirm viraemia.

HIV: HIV antibody tested using the Alere Determine HIV-1/2 rapid test (100% sensitive, 99.75% specific; Alere Medical, Chiba, Japan). Positive tests were double confirmed using a second test, the Vikia HIV 1/2 rapid test (100% sensitive, 99.5% specific; bioMerieux, Boxtel, Netherlands).

Participants were given the results of their screening tests at a second visit, offered post-test counselling, and appropriately referred for care.

### Participant questionnaire

The participant questionnaire was administered to participants during their first visit to the dermatology clinic. Trained interviewers conducted the study using REDCap software installed on mobile phones or tablet devices [20]. Data were captured using these devices and subsequently uploaded to a secure server. The questionnaires administered to the patients comprised two domains: (1) basic demographic data and (2) risk factors associated with viral infections. All the data were collected as categorical variables except for age which was continuous.

### Sampling approach

We undertook a sample size calculation based on the primary outcome viz. the proportion of HIV, HBV and FKN infections among African men. Findings from previous surveys in the Western Cape in South Africa reported prevalences of 15% for HIV and 8% for HBV among the population not considering FKN infection. The prevalence of FKN in men age > 18yrs is 10.5%. Considering an HIV prevalence of 22.5% in FKN group and 15% in the control group (odds ratio of 1.65) a total sample size of 855 (171 cases and 684 controls) was required to detect the difference between cases and controls using a chi-squared test with a 0.05 two-sided significance level and will have 80% power. The study adopted a convenience sampling of all the FKN infected patients visiting the dermatology clinic which were then matched with the age-appropriate controls.

### Remuneration

Participants were not remunerated for participation but were provided with a transport stipend to allow for the follow up visit.

### Ethics

The Human Research Ethics Committee of the University of Cape Town Faculty of Health Science (approval number 784/2015) approved the study.

### Statistical analysis

The data were analysed using STATA/IC version 16.0 (Stata Corp, College Station, TX). A composite variable of any viral infection (any *HBsAg or HCV antibody or HIV* infection) was used as the disaggregating variable for data presentation. Data were also stratified by a diagnosis of FKN, i.e., cases versus controls and geographical location (Mthatha and Cape Town). Continuous variables were expressed as medians (interquartile range, IQR) since the data were skewed. Proportions and percentages were used to describe categorical variables. The Wilcoxon rank-sum test for independent variables was used to compare the continuous data and the disaggregating variables.

The association between categorical variables was done using Pearson’s Chi-square test (χ 2) or Fisher’s Exact test as appropriate. Univariate and multivariate logistic regression were used to identify factors independently associated with the outcome of a viral infection, with a clean-shave hairstyle as a risk factor of interest, other covariates and age as a potential confounder. The analysis done was on a complete case analysis basis. A p-value of <0.20 in the univariate analysis was used to select the risk factors included in the multivariate model along with the risk factor of interest. The multivariate model results were expressed as adjusted odds ratios (aORs) and 95% confidence intervals (95% CIs). A p-value of less than or equal to 0.05 was used to denote statistical significance on interpreting associations. Missing data numbers are listed (S1 Fig).

## Results

### Participant characteristics

In total, 1163 men, median age 33.4 years [IQR 27.8–42.0], were evaluated (Table 1). The ratio of FKN cases to controls was 1:4, with a fractional proportion (0.5%, 6/1060) self-reporting any other current scalp ailment. Those seropositive for any of the screened for viral infections were significantly older than those without any infection, 37.1 years [IQR 31.0–44.4] vs. 32.3 years [IQR 26.9–41.0] p < 0.001, respectively. Of the participants, less than half were either married or co-habiting, and of these a significantly greater proportion were positive for any viral infection compared to those who were single, 43.3% vs. 33.1%; p < 0.01 respectively. Almost three-quarters, 73.4% (849/1156) of participants were fathers to one or more children and almost all, 91.2% (1057/1159) were circumcised, mostly traditionally performed.

**Table 1:** Characteristics of participants disaggregated by presence of any viral infection including Hepatitis B, Hepatitis C or HIV.

Variable	Total (n = 1,163)	Any viral infection (n = 254)	No viral infection (n = 909)	p-value
Age at presentation, years [median, IQR]	33.4 (27.8–42.0)	37.1 (31.0–44.4)	32.3 (26.9–41.0)	***<0.001***
Age categories				
20- < 40 years	821/1158 (70.9)	158/154 (62.2)	663/904 (73.3)	***<0.01***
40-to<60 years	308/1158 (26.6)	90/154 (35.4)	218/904 (24.1)	
≥ 60 years	29/1158 (2.5)	6/154 (2.4)	23/904 (2.5)	
Study group				
Cases	210/1,162 (18.1)	48/254 (18.9)	162/908 (17.8)	0.70
Controls	952/1,162 (81.9)	206/254 (81.1)	746/908 (82.2)	
Diagnosis of FKN (FKN+)	212/1,162 (18.2)	47/254 (18.5)	165/908 (18.2)	0.90
Ever had a clean haircut	1,107/1,160 (95.4)	246/254 (96.9)	861/906 (95.0)	0.22
Frequency of haircuts last month				
0	48/1,151 (4.2)	9/253 (3.6)	39/898(4.3)	0.22
1	249/1,151 (21.6)	42/253 (16.8)	207/898 (23.1)	
2	402/1,151 (35.0)	92/253 (36.0)	310/898 (34.5)	
3	110/1,151 (9.6)	26/253 (10.4)	84/898 (9.4)	
> 3	342/1,151 (29.7)	84/253 (33.2)	258/898 (28.7)	
Use clippers	834/1,163 (71.7)	172/254 (67.7)	662/909 (72.8)	0.11
Razor blade	326/1,163 (28.0)	88/254 (34.7)	238/909 (26.2)	***0.01***
Shaving cream	18/1,163 (1.6)	2/254 (0.8)	16/909 (1.8)	0.39
Scalp tender/sore after shave	592/1,147 (51.6)	130/253 (51.4)	462/894 (51.7)	0.93
Never bled after a shave	624/1,163 (53.7)	139/254 (54.7)	485/909 (53.4)	0.70
I sometimes bleed	498/1,163 (42.8)	101/254 (39.8)	397/909 (43.7)	0.27
I usually bleed	33/1,163 (2.8)	14/254 (5.5)	19/909 (2.1)	***<0.01***
How many children do you have				
0	307/1,156 (26.6)	51/253 (20.2)	256/903 (28.4)	***<0.01***
1-2	531/1,156 (45.9)	110/253 (43.5)	421/903 (46.6)	
> 2	318/1,156 (27.5)	92/253 (36.4)	226/903 (25.0)	
Married or co-habiting	408/1,156 (35.3)	109/252 (43.3)	299/904 (33.1)	***<0.01***
Circumcised	1,057/1,159 (91.2)	241/254 (94.9)	816/905 (90.2)	***0.02***
If circumcised (method)				
Traditional	950/1,055 (90.1)	224/241 (93.0)	726/814 (89.2)	0.09
Medical	105/1,055 (9.9)	17/241 (7.0)	88/814 (10.8)	
Sexual intercourse (penetrative)	1,010/1,160 (87.1)	224/252 (88.9)	786/908 (86.6)	0.33
Regular sexual partners				
1	857/1,135 (75.5)	193/250 (77.2)	664/885 (75.0)	0.63
2	210/1,135 (18.5)	45/250 (18.0)	165/885 (18.6)	
> 2	68/1,135 (6.0)	12/250 (4.8)	56/885 (6.3)	
Sex with person not your regular	461/1,142 (40.4)	105/252 (41.7)	356/890 (40.0)	0.63
Unprotected sex in the past month	646/1,141 (56.6)	131/251 (52.2)	515/890 (57.9)	0.11
Sores or genital discharge before	329/1,138 (71.1)	87/249 (34.9)	242/889 (27.2)	***0.02***
Ever had anal sex with a male	19/1,143 (1.7)	5/252 (2.0)	14/891 (1.6)	0.65
Ever injected drugs to get high	54/1,159 (4.7)	10/252 (4.0)	44/907 (4.8)	0.56
Smoked drugs for high/alcohol to be drunk	742/1,157 (64.1)	160/253 (63.2)	582/904 (64.4)	0.74
Used drugs/alcohol before sex	562/1,161 (48.4)	119/253 (47.0)	443/908 (48.8)	0.62
Vaccinated as a child				
Yes	877/1,151 (76.2)	205/250 (82.0)	672/901 (74.6)	***0.05***
Never	83/1,151 (7.2)	14/250 (5.6)	69/901 (7.7)	
Don’t know	191/1,151 (16.6)	31/250 (12.4)	160/901 (17.8)	
Ever received Hep B vaccination				
Yes	267/1,158 (23.1)	41/253 (16.2)	226/905 (25.0)	***0.01***
Never	492/1,158 (42.5)	123/253 (48.6)	369/905 (40.8)	
Don’t know	399/1,158 (34.4)	89/253 (35.2)	310/905 (34.2)	
Hep B boosters received				
0	25/267 (9.4)	5/41 (12.2)	20/226 (8.9)	0.89
1	69/267 (25.8)	11/41 (26.8)	58/226 (25.7)	
2	79/267 (29.6)	10/41 (24.4)	69/226 (30.5)	
> 2	43/267 (16.1)	6/41 0 (14.6)	37/226 (16.4)	
Don’t know	51/267 (19.1)	9/41 (22.0)	42/226 7 (18.6)	
Scarifications from healer	384/1,126 (34.1)	83/241 (34.4)	301/885 (34.0)	0.90
Share a toothbrush with partner	167/1,156 (14.5)	36/252 (14.3)	131/904 (14.5)	0.94
Tattoo/piercing informally done	212/1,157 (18.3)	58/252 (23.0)	154/905 (17.0)	***0.03***

*•Composite any viral infection – HBsAg positive, HCV antibody positive or HIV infection*

*•For each question, the total number equates to the question fully answered by a participant*

*•Data reported as n/N (%) for proportions and median (IQR) for continuous data.*

*•Associations between categories were tested using chi-square or Fishers exact tests and Mann Whitney test for continuous data*

Most of the participants, 95.4% (1107/1160), reported having “ever had a clean/shave haircut’ with 96.9% (246/254) of these having a seropositive result for any viral infection. Over a quarter, 28% (362/1163) of the participants reported, in addition to hair clipper use, they also received head shaving with a razor. Here, the risk of having any viral infection was significantly greater, 34.7% vs. 26.2%, p = 0.01. Probable previous sexually transmitted infections (genital lesions or penile discharge) were higher in those with any viral infection than those without, 34.9% vs. 27.2%, p = 0.02 respectively. 76.2% reported childhood vaccination being received, but overall rates of actual confirmed hepatitis B vaccination were low at 23.1%. In those with no viral infection, the rate of confirmed vaccination was higher, 25.0% vs. 16.2%; p = 0.01 respectively. Tattoos or other informal piercings were higher in those with any viral infection compared to those without:23.0%, 58/252 vs. 17%, 154/905; p = 0.03 respectively Table 1.

### Univariate analysis of risk factor characteristics

Regarding the 2 major study sites, participants from Cape Town tended to be older, than those from Mthatha, (34.7 years [IQR 28.7–43.0] vs. 31.0 years, [IQR 26.0–39.2], p < 0.001) respectively. More than a third (37.5%, 308/821) of the participants from Cape Town were either married or co-habiting while only 29.9% from Mthatha had the same partner status (p = 0.01). The circumcision rate in Mthatha compared to Cape Town, 96.1%, 323/336 vs. 89.2%, 734/823; p < 0.001 respectively, was greater. Of those diagnosed with FKN, a significantly greater number were from Mthatha than Cape Town, 25.4%, 85/335 vs. 15.4%, 127/827; p < 0.001, respectively (Table 2). A few participants in Cape Town (2.1%, 17/827) made use of a lubricating shaving cream, with only 0.3% (1/336) in Mthatha. In terms of associated extraneous factors for infection risk, substance use before sex was self-reported by more than half of the Cape Town participants with a third from Mthatha reporting use, 54.3%, 48/825 vs. 33.9%, 114/336; p < 0.001 respectively. A higher proportion of toothbrush sharing was recorded in Cape Town, 17.8%, 146/820 vs. 6.3%, 21/336; p < 0.001 in Mthatha. Tattoos or other informal piercings were more frequent in Cape Town compared to Mthatha participants, 19.7%, 162/821 vs. 14.9%, 50/336; p = 0.05 respectively Table 2. Table 3 denotes the overall frequencies of viral infections in the FKN and control groups, denoting no statistically significant difference on univariate analysis between the groups.

**Table 2:** Baseline characteristics of participants disaggregated by clinical FKN diagnosis and the study site.

Variable	Cape town			uMthatha			Total: Study sites			Total: Study group		
	Cases n=123	Controls n=704	p-value	Cases n=87	Controls n=248	p-value	Cape town n=827	uMthatha n=335	p-value	Cases n=210	Controls n=952	p-value
Age at presentation [IQR]	34.7 [28.1-41.1]	34.7 [28.7-43.4]	0.38	34.2 [27.8-42.8]	29.9 [25.6-38.0]	*<0.01*	34.7 [28.7-43.0]	31 [26.0-39.2]	*<0.001*	34.4 [28.1-42.1]	33.2 [27.7-41.9]	0.58
Age categories (%)												
20-<40 years	86/120 (71.7)	478/704 (67.9)	0.39	59/86 (68.6)	198/248 (79.8)	0.06	564/824 (68.5)	257/334 (77.0)	*0.02*	145/206 (70.4)	676/952 (71.0)	0.81
40-<60 years	33/120 (27.5)	204/704 (29.0)		24/86 (27.9)	47/248 /919.0)		237/824 (28.8)	71/334 (21.3)		57/206 (27.7)	251/952 (26.4)	
≥60 years	1/120 (0.8)	22/704 (3.1)		3/86 (3.5)	3/248 (1.2)		23/824 (2.8)	6/334 (1.8)		4/206 (1.9)	25/952 (2.6)	
Diagnosis of FKN (FKN+)	118/123 (95.9)	9/704 (1.3)	*<0.001*	82/87 (94.3)	3/248 (1.2)	*<0.001*	127/827 (15.4)	85/335 (25.4)	*<0.001*	200/210 (95.2)	12/952 (1.3)	*<0.001*
Healthy looking scalp (FKN-)	4/5 (80.0)	693/695 (99.7)	*0.02*	1/5 (20.0)	243/245 (99.2)	*<0.001*	697/700 (99.6)	244/250 (97.6)	*0.01*	5/10 (50.0)	936/940 (99.6)	*<0.001*
Any other scalp ailment	1/87 (0.9)	2/247 (0.3)	0.35	1/87 (1.2)	2/247 (0.8)	1	3/826 (0.4)	3/334 (0.9)	0.36	2/209 (1.0)	4/951 (0.4)	0.3
Ever had a clean-shave haircut	116/121 (95.9)	666/703 (94.7)	0.6	83/87 (95.4)	241/248 (97.2)	0.42	782/824 (94.9)	325/336 (96.7)	0.18	199/208 (95.7)	907/951 (95.4)	0.85
Number of haircuts last month												
0	4/120 (3.3)	28/695 (4.0)	0.11	1/87 (1.2)	15/248 (6.1)	*<0.001*	32/815 (3.9)	16/336 (4.8)	0.14	5/207 (2.4)	43/943 (4.6)	*<0.001*
1	24/120 (20.0)	159/695 (22.9)		15/87 (17.2)	51/248 (20.6)		183/815 (22.6)	66/336 (19.6)		39/207 (18.8)	210/943 (22.3)	
2	33/120 (27.5)	255/695 (36.7)		17/87 (19.5)	96/248 (38.7)		288/815 (35.6)	114/336 (33.9)		50/207 (24.2)	351/943 (37.2)	
3	15/120 (12.5)	70/695 (10.1)		8/87 (9.2)	17/248 (6.9)		85/815 (10)	25/336 (7.4)		23/207 (11.1)	87/943 (9.2)	
>3	44/120 (36.7)	183/695 (26.3)		46/87 (52.9)	69/248 (27.8)		227/815 (27.9)	115/336 (34.2)		90/207 (43.5)	252/943 (26.7)	
Used clippers	85/123 (69.1)	501/704 (71.2)	0.64	54/87 (62.1)	193/248 (77.8)	*<0.01*	586/827 (70.9)	248/336 (73.8)	0.31	139/210 (66.2)	694/952 (72.9)	*0.05*
Razor blade	35/123 (28.5)	196/704 (27.8)	0.89	36/87 (41.4)	59/248 (23.8)	*<0.01*	231/827 (27.9)	95/336 (28.3)	0.91	71/210 (33.8)	255/952 (26.8)	*0.04*
Shaving cream	4/123 (3.3)	13/704 (1.9)	0.31	0	1/248 (0.4)	1	17/827 (2.1)	1/336 (0.3)	*0.03*	4/210 (1.9)	14/952 (1.5)	0.65
Calp tender/sore after shave	74/120 (61.7)	328/693 (47.3)	*<0.01*	40/86 (46.5)	112/247 (45.3)	0.85	402/813 (49.5)	153/334 (45.8)	0.27	114/206 (55.3)	440/940 (46.8)	*0.03*
Never bled after a shave	41/123 (33.3)	386/704 (54.8)	*<0.001*	39/87 (44.8)	158/248 (63.7)	*<0.01*	427/827 (51.6)	197/336 (58.6)	*0.04*	80/210 (38.1)	544/952 (57.1)	*<0.001*
I sometimes bleed	71/123 (57.7)	298/704 (42.3)	*<0.01*	44/87 (50.6)	84/248 (33.9)	*0.01*	369/827 (44.6)	129/336 (38.4)	0.06	115/210 (54.8)	382/952 (40.1)	*<0.001*
I usually bleed	8/123 (6.5)	15/704 (2.1)	*0.01*	4/87 (4.6)	6/248 (2.4)	0.3	23/827 (2.8)	10/336 (3.0)	0.85	12/210 (5.7)	21/952 (2.2)	*0.01*
Number of children												
0	31/120 (25.8)	177/700 (25.3)	0.98	21/87 (24.2)	78/248 (31.4)	0.17	208/820 (25.4)	99/336 (29.5)	0.17	52/207 (25.1)	255/948 (26.9)	0.67
1-2	54/120 (45.0)	321/700 (45.9)		39/87 (44.8)	116/248 (46.8)		375/820 (45.7)	156/336 (46.4)		93/207 (44.9)	437/948 (46.1)	
>2	35/120 (29.2)	202/700 (28.9)		27/87 (31)	54/248 (21.8)		237/820 (28.9)	81/336 (24.1)		62/207 (30.0)	256/948 (27.0)	
Married or co-habiting	52/121 (43.0)	256/700 (36.6)	0.18	33/87 (37.9)	67/247 (27.1)	0.06	308/821 (37.5)	100/335 (29.9)	*0.01*	85/208 (40.9)	323/947 (34.1)	0.07
Circumcised	111/121 (91.7)	623/702 (88.8)	0.33	83/87 (95.4)	239/248 (96.4)	0.69	734/823 (89.2)	323/336 (96.1)	*<0.001*	194/208 (93.3)	862/950 (90.7)	0.24
If circumcised (method)												
Traditional	98/111 (88.3)	557/621 (89.6)	0.66	75/83 (90.4)	219/239 (91.6)	0.72	655/732 (89.5)	295/323 (91.3)	0.36	173/194 (89.2)	776/860 (90.2)	0.66
Medical	13/111 (11.7)	64/621 (10.4)		8/83 (9.6)	20/239 (8.4)		77/732 (10.5)	28/323 (8.7)		21/194 (10.8)	84/860 (9.8)	
Sexual intercourse (penetrative)	106/122 (86.9)	605/703 (86.1)	0.81	74/87 (85.1)	224/247 (90.7)	0.15	711/825 (86.2)	299/335 (89.3)	0.16	180/209 (86.1)	829/950 (87.3)	0.66
Regular sexual partners												
1	90/119 (75.6)	533/688 (77.5)	0.87	60/83 (72.3)	173/244 (70.9)	0.09	623/807 (77.2)	234/328 (71.3)	0.1	150/202 (74.3)	706/932 (75.8)	0.27
2	21/119 (17.7)	116/688 (16.9)		14/83 (16.9)	59/244 (24.2)		137/807 (17.0)	73/328 (22.3)		35/202 (17.3)	175/932 (18.8)	
>2	8/119 (6.7)	39/688 (5.7)		9/83 (10.8)	12/244 (4.9)		47/807 (5.8)	21/328 (6.4)		17/202 (8.4)	51/932 (5.5)	
Sex with not regular person												
Yes	50/120 (41.7)	279/692 (40.3)	0.78	26/84 (31.0)	105/245 (42.9)	*0.05*	329/812 (40.5)	132/330 (40.0)	0.87	76/204 (37.3)	384/937 (41.0)	0.33
Never	70/120 (58.3)	413/692 (59.7)		58/84 (69.0)	140/245 (57.1)		483/812 (59.5)	198/330 (60.0)		128/204 (62.7)	554/937 (59.0)	
Unprotected sex	78/120 (65.0)	392/691 (56.7)	0.09	50/83 (60.2)	125/246 (50.8)	0.14	470/811 (58.0)	176/330 (53.3)	0.15	128/203 (63.1)	517/937 (55.2)	*0.04*
Sores/genital discharge before												
Yes	36/119 (30.3)	196/690 (28.4)	0.68	28/83 (33.7)	69/245 (28.2)	0.34	232/809 (28.7)	97/329 (29.5)	0.79	64/202 (31.7)	265/935 (28.3)	0.34
Never	83/119 (69.7)	494/690 (71.6)		55/83 (66.3)	176/245 (71.8)		577/809 (71.3)	232/329 (70.5)		138/202 (68.3)	670/935 (71.7)	
Ever injected drugs	5/122 (4.1)	34/702 (4.8)	0.72	3/86 (3.5)	12/248 (4.8)	0.6	39/824 (4.7)	15/335 (4.5)	0.85	8/208 (3.9)	46/950 (4.8)	0.54
Smoked drugs/Alcohol	80/122 (65.6)	454/702 (64.7)	0.85	49/87 (56.3)	158/245 (64.5)	0.18	534/824 (64.8)	208/333 (62.5)	0.45	129/209 (61.7)	612/947 (64.6)	0.43
Used drugs before sex	63/122 (51.6)	385/703 (54.8)	0.52	28/87 (32.2)	85/248 (34.3)	0.72	448/825 (54.3)	114/336 (33.9)	*<0.001*	91/209 (43.5)	470/951 (49.4)	0.12
Vaccinated as a child												
Yes	87/122 (71.3)	526/694 (75.8)	0.54	63/87 (72.4)	200/247 (81.0)	*0.02*	613/816 (75.1)	264/335 (78.8)	0.32	150/209 (71.6)	726/941 (77.2)	0.16
Never	11/122 (9.0)	48/694 (6.9)		4/87 (4.6)	20/247 (8.1)		59/816 (7.2)	24/335 (7.2)		15/209 (7.2)	68/941 (7.2)	
Don’t know	24/122 (19.7)	120/694 (17.3)		20/87 (23.0)	27/247 (10.9)		144/816 (17.7)	47/335 (14.0)		44/209 (21.2)	147/941 (15.6)	
Confirmed ever received Hep B vaccination												
Yes	18/122 (14.8)	172/701 (24.6)	0.06	9/86 (10.4)	68/248 (27.4)	*0.01*	190/823 (23.1)	77/335 (23.0)	*0.05*	27/208 (13.0)	240/949 (25.3)	*<0.01*
Never	59/122 (48.4)	307/701 (44.1)		39/86 (45.4)	87/248 (35.1)		366/823 (44.5)	126/335 (37.6)		98/208 (46.9)	394/949 (41.5)	
Don’t know	45/122 (36.9)	222/701 (31.3)		38/86 (44.2)	93/248 (37.5)		267/823 (32.4)	132/335 (39.4)		83/208 (40.1)	315/949 (33.2)	
Hep B boosters received												
0	0	12/172 (7.0)	0.71	1/9 (11.1)	12/68 (17.6)	*<0.01*	235/823 (28.6)	95/336 (28.3)	*<0.01*	60/209 (28.4)	270/949 (28.5)	0.09
1	4/18 (22.2)	43/172 (25.0)		1/9 (11.1)	21/68 (30.9)		101/823 (12.3)	35/336 (10.4)		19/209 (9.1)	117/949 (12.3)	
2	6/18 (33.3)	58/172 (33.7)		1/9 (11.1)	14/68 (20.6)		96/823 (11.7)	18/336 (5.3)		12/209 (5.8)	102/949 (10.7)	
>2	5/18 (27.8)	31/172 (18.0)		4/9 (44.5)	3/68 (4.4)		46/823 (5.6)	11/336 (3.3)		11/209 (5.3)	46/949 (4.8)	
Don’t know	3/18 (16.7)	28/172 (16.3)		2/9 (22.2)	18/68 (26.5)		345/823 (41.9)	177/336 (52.7)		107/209 (51.4)	414/949 (43.6)	
Scarification from healer (10yrs)	40/116 (34.5)	231/7674 (34.3)	0.97	36/87 (41.4)	77/248 (31.1)	0.08	271/790 (34.3)	113/336 (33.6)	0.83	76/203 (37.4)	308/922 (33.4)	0.27
Share a toothbrush	22/121 (18.2)	124/699 (17.7)	0.91	5/87 (5.8)	16/248 (6.5)	0.82	146/820 (17.8)	21/336 (6.3)	*<0.001*	27/208 (13.0)	140/947 (14.8)	0.5
Tattoo/piercing informally done	22/122 (18.0)	140/699 (20.0)	0.61	11/87 (12.6)	39/248 (15.7)	0.49	162/821 (19.7)	50/336 (14.9)	*0.05*	33/209 (15.8)	179/947 (18.9)	0.29

*• Composite any viral infection – HBsAg positive, HCV antibody positive or HIV infection*

*• For each question, the total number equates to the question fully answered by a participant*

*• Data reported as n/N (%) for proportions and median (IQR) for continuous data.*

*• Associations between categories were tested using chi-square or Fishers exact tests and Mann Whitney test for continuous data*

**Table 3:** Association between clinical FKN diagnosis and viral infection or co-infection amongst all participants and those with healthy scalps.

Variable	Combined			
	Total (n, %)	FKN patients	Healthy controls	p-value
HIV infection	200/1163 (17.2)	41/210 (19.5)	159/953 (16.7)	0.33
HBV infection	80/1163 (6.9)	13/210 (6.2)	67/953 (7.1)	0.66
HCV infection	5/1163 (0.4)	0/210	5/953 (0.5)	0.29
HIV/HBV co-infection	28/1161 (2.4)	6/210 (2.9)	22/953 (2.3)	0.64
HIV/HCV co-infection	2/1157 (0.2)	0/210	2/953 (0.2)	0.51

Participants with FKN had more frequent clean-shave haircuts (>3 in the past month) compared to controls, 43.5%, 90/207 vs. 26.7%, 252/943; p < 0.001. Those with FKN reported using a razor blade more frequently, 33.8%, 71/210 vs. 26.8%, 255/952; p = 0.04 (Table 2). A history of sometimes bleeding (54.8%, 115/210) or always bleeding (5.7%, 12/210) in those with FKN, when compared to controls, was significantly greater, 46.8%, 440/940; 40.1%, 382/952; 2.2%, 21/952; p = 0.03, < 0.001 & 0.01, respectively. A greater proportion of reported unprotected sex amongst those with FKN compared to controls, 63.1%, 128/203 vs. 55.2%, 517/937; p = 0.04 respectively, was observed while fewer hepatitis B vaccination was reported in the FKN patients compared to controls, 13.0%, 27/208 vs. 25.3%, 240/949; p < 0.01, respectively. The most pertinent risk of interest viz. having ever had a clean-shave haircut, was positively associated with having any viral infection or coinfection, but the associations were not statistically significant Table 3.

### Multivariate analyses – main outcomes

On multivariate analysis (Table 4) those who usually bled after or during a clean-shave haircut, aOR = 2.51 [95% CI 1.16–5.42], p = 0.02; those who have had sexually transmitted sores or a genital discharge, aOR = 1.58 [95% CI 1.13–2.22], p = 0.01 and the participants who had a tattoo/piercing informally done, aOR=1.84 [95% CI 1.13–2.22], p = 0.01 had greater likelihood of being HIV seropositive compared to those that did not Table 4. The multivariate analysis for a seropositive HBV and HCV outcome after adjusting for ever having had a clean haircut yielded no statistically significant increase or decrease in odds for the considered covariates Table 5 **and** 6. We found similar associations between HIV/HBV or HIV/HCV co-infections and risk factors identified in the univariate analysis as those observed in the HIV mono-infection multivariate analysis Table 7.

**Table 4:** Multivariate analysis of cross-sectional data as risk factors of viral infections or co-infections.

Variable	HIV		HBV		HCV		HIV/HBV		HIV/HCV	
	aOR (95% CI)	p-value	aOR (95% CI)	p-value	aOR (95% CI)	p-value	aOR (95% CI)	p-value	A OR (95% CI)	p-value
Ever had a clean haircut										
No	*Ref*		*Ref*		*Ref*		*Ref*		*Ref*	
Yes	1.69 (0.64-4.49)	0.29	2.02 (0.47-8.62)	0.34	-	-	1.59 (0.69-3.68)	0.27	1.73 (0.66-4.59)	0.27
Age at presentation	1.02 (1.00-1.04)	0.07			1.05 (0.98-1.12)	0.15	1.01 (1.00-1.03)	0.09	1.02 (1.00-1.04)	***0.05***
Use clippers										
No	*Ref*		*Ref*				*Ref*		*Ref*	
Yes	1.78 (0.76-4.19)	0.18	0.72 (0.22-2.33)	0.59			1.20 (0.55-2.62)	0.65	1.76 (0.75-4.10)	0.19
Razor blade										
No	*Ref*		*Ref*				*Ref*		*Ref*	
Yes	2.01 (0.86-4.69)	0.11	1.11 (0.34-3.59)	0.86			1.30 (0.60-2.85)	0.51	1.97 (0.85-4.58)	0.11
I usually bleed										
No	*Ref*						*Ref*		*Ref*	
Yes	2.51 (1.16-5.42)	***0.02***					2.20 (1.04-4.63)	***0.04***	2.43 (1.13-5.25)	***0.02***
How many children do you have										
0	*Ref*						*Ref*		*Ref*	
1-2	0.95 (0.61-1.49)	0.82					0.96 (0.64-1.43)	0.84	0.98 (0.63-1.53)	0.92
>2	1.37 (0.81-2.31)	0.24					1.31 (0.81-2.10)	0.27	1.40 (0.83-2.35)	0.21
Married or co-habiting										
No	*Ref*						*Ref*		*Ref*	
Yes	1.37 (0.94-2.01)	0.1					1.32 (0.93-1.86)	0.12	1.34 (0.92-1.95)	0.13
Circumcised										
No	*Ref*						*Ref*		*Ref*	
Yes	1.68 (0.84-3.37)	0.14					1.68 (0.90-3.12)	0.1	1.69 (0.84-3.39)	0.14
Unprotected sex in the past month										
No	*Ref*						*Ref*		*Ref*	
Yes	0.63 (0.46-0.88)	***0.01***					0.71 (0.53-0.96)	***0.03***	0.66 (0.48-0.91)	***0.01***
Sores or genital discharge before										
No	*Ref*						*Ref*		*Ref*	
Yes	1.58 (1.13-2.22)	***0.01***					1.46 (1.07-1.20)	***0.02***	1.58 (1.13-2.21)	***0.01***
Tattoo/piercing informally done										
No	*Ref*						*Ref*		*Ref*	
Yes	1.84 (1.26-2.71)	***<0.01***					1.58 (1.10-2.27)	***0.01***	1.85 (1.27-2.72)	***<0.01***
Smoked drugs for high/alcohol to be drunk										
No					Ref					
Yes					0.16 (0.02-1.51)	0.11				

**Table 5:** HIV and HBV rates in varying risk of injury with a clean shave cut in those with a clinical FKN diagnosis.

Injury	Never bleed	I sometimes bleed	I always bleed	p-value
HIV positive	13/80 (16.3)	21/115 (18.3)	7/12 (58.3)	***0.02***
HIV negative	67/80 (83.7)	94/115 (81.7)	5/12 (41.7)	
HBV positive	5/80 (6.3)	7/115 (6.1)	1/12 (8.3)	0.89
HBV negative	75/80 (93.7)	108/115 (93.9)	11/12 (91.7)	

**Table 6:** Univariate analysis of a clean-hair style as a risk factor of viral infection or co-infections.

Variable	Univariate analysis	
	**Odds Ratio (95% CI**	**p-value**
HIV + vs. HIV-	1.7 (0.7-3.9)	0.25
HBV + vs. HBV-	1.9 (0.5-8.1)	0.37
HCV + vs. HCV-	1.0	–
HIV/HBV + vs. HIV/HBV-	1.6 (0.7-3.4)	0.23
HIV/HCV + vs. HIV/HCV-	1.7 (0.7-4.0)	0.23

**Table 7:** Univariate analysis of survey data as risk factors of viral infection or co-infections.

Variable	HIV		HBV		HCV		HIV/HBV		HIV/HCV	
	OR (95% CI)	p-value	OR (95% CI)	p-value	OR (95% CI)	p-value	OR (95% CI)	p-value	OR (95% CI)	p-value
Age at presentation	1.03 (1.02-1.04	***<0.001***	1.01 (0.99-1.03)	0.33	1.06 (0.99-1.14)	0.08	1.03 (1.01-1.04)	***<0.001***	1.03 (1.02-1.04)	***<0.001***
Age categories										
20-<40 years	*Ref*		*Ref*		*Ref*		*Ref*		*Ref*	
40-to<60 years	1.90 (1.37-2.63)	***<0.001***	1.23 (0.75-2.01)	0.41	2.68 (0.38-19.08	0.33	1.71 (1.27-2.32)	***<0.001***	1.93 (1.40-2.67)	***<0.001***
≥60 years	1.54 (0.61-3.86)	0.36	-	-	14.63 (1.29-166.10)	***0.03***	1.10 (0.44-2.75)	0.84	1.51 (0.60-3.79)	0.38
Study group										
Cases	*Ref*		*Ref*		*Ref*		*Ref*		*Ref*	
Controls	0.83 (0.56-1.21)	0.33	1.15 (0.62-2.12)	0.66	-	-	0.92 (0.64-1.32)	0.66	0.84 (0.57-1.23)	0.37
Diagnosis of FKN (FKN+)										
No	*Ref*		*Ref*		*Ref*		*Ref*		*Ref*	
Yes	1.19 (0.81-1.75)	0.36	0.78 (0.41-1.47)	0.44	-	-	1.03 (0.72-1.48)	0.86	1.18 (0.80-1.72)	0.41
Ever had a clean haircut										
No	*Ref*		*Ref*		*Ref*		*Ref*		*Ref*	
Yes	1.66 (0.70-3.95)	0.25	1.93 (0.46-8.09)	0.37	-	-	1.59 (0.74-3.42)	0.23	1.71 (0.72-4.04)	0.23
Frequency of haircuts last month										
0	*Ref*		*Ref*		*Ref*		*Ref*		*Ref*	
1	0.59 (0.26-1.35)	0.21	1.27 (0.28-5.80)	0.76	-	-	0.88 (0.40-1.95)	0.75	0.59 (0.26-1.35)	0.21
2	0.95 (0.44-2.04)	0.89	1.59 (0.37-6.92)	0.54	-	-	1.25 (0.59-2.69)	0.56	1.00 (0.46-2.15)	1
3	0.90 (0.38-2.18)	0.82	2.56 (0.54-12.00)	0.23	-	-	1.34 (0.57-3.13)	0.5	0.90 (0.38-2.18)	0.82
>3	1.12 (0.52-2.41)	0.78	1.97 (0.28-5.80)	0.37	-	-	1.41 (0.66-3.03)	0.38	1.13 (0.52-2.44)	0.76
Use clippers										
No	*Ref*		*Ref*		*Ref*		*Ref*		*Ref*	*** ***
Yes	0.72 (0.52-0.99)	***0.05***	0.67 (0.42-1.08)	0.1	1.58 (0.18-14.20)	0.68	0.77 (0.57-1.04)	0.09	0.73 (0.53-1.00)	***0.05***
Razor blade										
No	*Ref*		*Ref*		*Ref*		*Ref*		*Ref*	
Yes	1.75 (1.27-2.41)	***<0.01***	1.50 (0.94-2.42)	0.09	0.64 (0.07-5.75)	0.69	1.52 (1.12-2.04)	***<0.01***	1.73 (1.26-2.38)	***<0.01***
Shaving cream										
No	*Ref*		*Ref*		*Ref*		*Ref*		*Ref*	
Yes	0.28 (0.04-2.11)	0.22	0.79 (0.10-6.04)	0.82	-	-	0.45 (0.10-1.96)	0.29	0.27 (0.04-2.06)	0.21
Scalp tender/sore after shave										
No	*Ref*		*Ref*		*Ref*		*Ref*		*Ref*	
Yes	0.95 (0.70-1.29)	0.73	1.01 (0.64-1.60)	0.96	1.41 (0.23-8.46)	0.71	0.97 (0.73-1.28)	0.84	0.96 (0.71-1.30)	0.79
Never bled after a shave										
No	*Ref*		*Ref*		*Ref*		*Ref*		*Ref*	
Yes	1.04 (0.77-1.41)	0.79	0.90 (0.57-1.42)	0.66	-	-	1.04 (0.79-1.38)	0.79	1.08 (0.80-1.47)	0.61
I sometimes bleed										
No	*Ref*		*Ref*		*Ref*		*Ref*		*Ref*	
Yes	0.87 (0.64-1.19)	0.38	0.99 (0.62-1.56)	0.95	-	-	0.86 (0.65-1.15)	0.31	0.84 (0.62-1.14)	0.27
I usually bleed										
No	*Ref*		*Ref*		*Ref*		*Ref*		*Ref*	
Yes	2.86 (1.38-5.92)	***0.01***	1.91 (0.66-5.58)	0.24	-	-	2.76 (1.36-5.58)	***<0.01***	2.79 (1.35-5.78)	***<0.01***
How many children do you have										
0	Ref		*Ref*		*Ref*		*Ref*		*Ref*	
1-2	1.33 (0.88-2.01)	0.18	1.31 (0.73-2.37)	0.36	-	-	1.29 (0.89-1.86)	0.17	1.36 (0.90-2.06)	0.14
>2	2.26 (1.48-3.46)	***<0.001***	1.39 (0.73-2.65)	0.31	-	-	2.00 (1.36-2.95)	***<0.001***	2.29 (1.50-3.51)	***<0.001***
Married or co-habiting										
No	*Ref*		*Ref*		*Ref*		*Ref*		*Ref*	*** ***
Yes	1.66 (1.22-2.26)	***<0.01***	1.03 (0.64-1.66)	0.91	0.46 (0.05-4.10)	0.48	1.53 (1.15-2.04)	***<0.01***	1.63 (1.20-2.22)	***<0.01***
Circumcised										
No	*Ref*		*Ref*		*Ref*		*Ref*		*Ref*	*** ***
Yes	2.02 (1.03-3.94)	***0.04***	2.59 (0.80-8.37)	0.11	-	-	1.98 (1.09-3.60)	***0.03***	2.04 (1.04-4.00)	***0.04***
If circumcised (method)										
Traditional	*Ref*		*Ref*		*Ref*		*Ref*		*Ref*	
Medical	0.62 (0.34-1.13)	0.12	0.75 (0.32-1.77)	0.51	2.27 (0.25-20.54)	0.46	0.63(0.37-1.09)	0.1	0.66 (0.37-1.18)	0.16
Sexual intercourse (penetrative)										
No	*Ref*		*Ref*		*Ref*		*Ref*		*Ref*	
Yes	1.31 (0.80-2.13)	0.29	1.36 (0.64-2.89)	0.42	-	-	1.23 (0.79-1.90)	0.36	1.34 (0.82-2.18)	0.24
Regular sexual partners										
1	*Ref*		*Ref*		*Ref*		*Ref*		*Ref*	
2	1.00 (0.67-1.49)	1	0.68 (0.35-1.32)	0.26	-	-	0.95 (0.66-1.37)	0.78	0.98 (0.66-1.45)	0.91
>2	0.62 (0.29-1.33)	0.22	0.98 (0.38-2.53)	0.97	-	-	0.75 (0.39-1.42)	0.37	0.61 (0.28-1.29)	0.19
Sex with person not your regular										
No	*Ref*		*Ref*		*Ref*		*Ref*		*Ref*	
Yes	0.98 (0.71-1.34)	0.88	1.44 (0.91-2.27)	0.12	-	-	1.09 (0.82-1.45)	0.54	0.96 (0.70-1.31)	0.78
Unprotected sex in the past month										
No	*Ref*	*** ***	*Ref*		*Ref*		*Ref*		*Ref*	
Yes	0.73 (0.54-0.99	***0.05***	0.98 (0.62-1.56)	0.95	-	-	0.78 (0.59-1.03)	0.08	0.76 (0.56-1.03)	0.07
Sores or genital discharge before										
No	*Ref*	*** ***	*Ref*		*Ref*		*Ref*		*Ref*	
Yes	1.59 (1.15-2.20)	***0.01***	0.95 (0.57-1.57)	0.83	0.61 (0.07-5.51)	0.66	1.46 (1.08-1.97)	***0.01***	1.59 (1.16-2.20)	***0.01***
Ever had anal sex with a male										
No	*Ref*		*Ref*		*Ref*		*Ref*		*Ref*	
Yes	1.72 (0.61-4.84)	0.3	1	-	-	-	1.28 (0.46-3.59)	0.64	1.68 (0.60-4.72)	0.32
Ever injected drugs to get high										
No	*Ref*		*Ref*		* *		*Ref*		*Ref*	
Yes	0.97 (0.47	0.93	0.25 (0.03-1.80)	0.17	*Ref*	-	0.82 (0.41-1.65)	0.58	0.95 (0.45-1.97)	0.88
Smoked drugs for high/alcohol to be drunk										
No	*Ref*		*Ref*		*Ref*		*Ref*		*Ref*	
Yes	0.93 (0.68-1.28)	0.67	1.11 (0.68-1.79)	0.68	0.14 (0.02-1.24)	0.08	0.96 (0.72-1.28)	0.78	0.91 (0.66-1.24)	0.54
Used drugs/alcohol before sex										
No	*Ref*		*Ref*		*Ref*		*Ref*		*Ref*	
Yes	0.92 (0.68-1.25	0.6	1.19 (0.76-1.88)	0.45	-	-	0.95 (0.72-1.26)	0.73	0.90 (0.66-1.22)	0.49
Vaccinated as a child										
No	*Ref*		*Ref*		*Ref*		*Ref*		*Ref*	
Yes	1.32 (0.70-2.49)	0.39	3.35 (0.81-13.93)	0.1	-	-	1.48 (0.82-2.70)	0.19	1.35 (0.72-2.56)	0.35
Don’t know	0.89 (0.42-1.87)	0.76	2.24 (0.48-10.44)	0.31	-	-	0.95 (0.48-1.91)	0.9	0.90 (0.43-1.88)	0.77
Ever received Hep B vaccination										
No	*Ref*		*Ref*		*Ref*		*Ref*		*Ref*	
Yes	0.59 (0.39-0.91)	***0.02***	0.60 (0.30-1.17)	0.13	0.92 (0.08-10.20)	0.95	0.54 (0.37-0.80)	***<0.01***	0.59 (0.38-0.89)	***0.01***
Don’t know	0.84 (0.59-1.18)	0.31	1.10 (0.67-1.81)	0.69	1.23 (0.17-8.80)	0.83	0.83 (0.61-1.14)	0.26	0.86 (0.61-1.20)	0.37
Hep B boosters received										
0	*Ref*		*Ref*		*Ref*		*Ref*		*Ref*	
1	0.69 (0.19-2.52)	0.57	1.48 (0.16-13.88)	0.73	-	-	0.76 (0.23-2.45)	0.64	0.69 (0.19-2.52)	0.57
2	0.68 (0.19-2.42)	0.55	0.62 (0.05-7.18)	0.71	-	-	0.58 (0.18-1.89)	0.37	0.68 (0.19-2.45)	0.56
>2	0.69 (0.17-2.85)	0.61	1.17 (0.10-13.60)	0.9			0.65 (0.18-2.39)	0.52	0.69 (0.17-2.85)	0.61
Don’t know	0.98 (0.26-3.62)	0.97	1.50 (0.15-15.20)	0.73	-	-	0.86 (0.25-2.89)	0.8	0.98 (0.26-3.62)	0.97
Scarification from healer										
No	*Ref*		*Ref*		*Ref*		*Ref*		*Ref*	
Yes	1.09 (0.79-1.51)	0.6	0.94 (0.57-1.56)	0.82	0.48 (0.05-4.32)	0.51	1.03 (0.77-1.40)	0.83	01.05 (0.77-1.46)	0.74
Share a toothbrush with partner										
No	*Ref*		*Ref*		*Ref*		*Ref*		*Ref*	
Yes	0.88 (0.56-1.37)	0.56	1.28 (0.70-2.34)	0.42	-	-	0.99 (0.67-1.48)	0.98	0.85 (0.54-1.34)	0.49
Tattoo/piercing informally done										
No	*Ref*		*Ref*		*Ref*		*Ref*		*Ref*	
Yes	1.72 (1.20-2.47)	***<0.01***	1.12 (0.64-1.20)	0.69	1.11 (0.12-10.03)	0.92	1.49 (1.06-2.09)	***0.02***	1.74 (1.21-2.45)	***<0.01***

### Multivariate analyses—co-infection

In the analysis after adjusting for ever having had a close-shave haircut, the odds of being HIV/HBV or HIV/HCV co-infected increased with increasing age, however this did not reach significance for the former, aOR = 1.01 [95% CI 1.00–1.03], p = 0.09 but did for the latter aOR=1.02 [95% CI 1.00–1.04], p = 0.05. Those who usually bled after or during a clean-shave haircut, OR = a2.20 [95% CI 1.04–4.63], p = 0.04 those who had a sexually transmitted genital lesion or a penile discharge, aOR = 1.46 [95% CI 1.07–1.20], p = 0.02 and the participants who had a tattoo/piercing informally done, aOR = 1.58 [95% CI 1.10–2.27], p = 0.01 had a greater likelihood of being HIV/HBV coinfected compared to those that did not.

Those who usually bleed after or during a clean-shave haircut, aOR = 2.43 [95% CI 1.13–5.25], p = 0.02, those who have had sexually transmitted sores or a genital discharge, aOR = 1.58 [95% CI 1.13–2.21], p = 0.01 and the participants who had a tattoo/piercing informally done, aOR = 1.85 [95% CI 1.27–2.72], p < 0.01 had a greater risk odds of being HIV/HCV coinfected compared to those that did not.

When analysing the cohort for having ever had a close-shave cut, 95.4% reported having ever had a clean-shave haircut with 28% reporting additionally shaving their heads with a razor. In this sub-group, the risk of having any viral infection was significantly greater, 34.7% vs. 26.2%, p = 0.01. This does suggest that using a razor, with bleeding does impart risk, although there are many confounding variables. An interesting aspect is that despite the high background rate of circumcision, a reported HIV protective factor, HIV seroprevalence rates remain high in this cohort. It does suggest that other factors override the potential value of circumcision in HIV prevention in this group.

## Discussion

Our study aimed to determine HIV, HBV, and HCV seroprevalence in those with and without a unique hairstyle in South Africa, and potentially transversal to other styles of haircutting where bleeding risk is involved. The close-shave style or “chiskop” enables potential transmission risk given its propensity to produce *folliculitis keloidalis nuchea* or FKN. The bleeding associated with this dermatological condition and the use of clippers with or without a blade razor, enables the risk. Overall, we found no certain association between this haircut style and increased bloodborne infection risk.

The study population was young, median age of 33.4 [27.8–42.0] years. In terms of hepatitis B risk, during the period the study was conducted and given median age, many participants were born before April 1995. This date marks the introduction of the hepatitis B vaccine into the expanded programme of immunization in South Africa [21]. Rollout of the vaccine into all parts of the country was incremental, there was no catch-up or birth dose programme and full 3-dose vaccination, currently at 85%, took time to achieve. The effect is that participants in this study may thus be at risk of hepatitis B infection given non-immunity. However, in sub-Saharan Africa, the risk of hepatitis B transmission predominates in early childhood, with exposure occurring early. This suggests that some participants either have chronic established infection or previous exposure with HBsAg seroclearance. Even so, our overall 6.9% HBsAg seropositivity rate in our participants is above the current estimated HBsAg seroprevalence in South Africa. Based on modelling data from the Polaris observatory, during the study period, the estimated range of HBsAg positivity in South Africa was 4.8–5.1% [2]. Our data however did not find any correlation between FKN and risk of HBV, on both univariate and multivariate analysis. Equally HCV risk was not associated with FKN, and overall prevalence of HCV was low at <0.5%, in keeping with what is known in the general population in South Africa.

Greatest HCV risk lies within key populations such as people who inject drugs, a population not directly accounted for in this cohort as evidence by admitted substance use of a mostly non-injecting nature. It is conceivable that if participants with FKN from key HCV populations were more represented in the cohort, HCV rates may well be higher in the FKN participants. However, HIV-HCV co-infection risk was significantly associated with this cohort. Rates of co-infection of HIV-HCV in South Africa are increasing in key populations, notably people who inject drugs [22]. This is an aspect we have not directly considered in our study.

HIV seroprevalence was 17.2% overall, with no difference observed in those with FKN or healthy controls. The HIV seroprevalence rate remains concerningly high but is in keeping with national seroprevalence figures for a male cohort of the age range studied [1]. HIV in sub-Saharan Africa is a mostly heterosexually transmitted infection; however other risk transmission factors are plenty as was noted in the confidential questionnaire responses [23]. Even though FKN did not predict for HIV seropositivity, multivariate analysis confirmed that the odds of HIV increased with increasing age, although not significantly so, OR = 1.02 [95% CI 1.00–1.04]; p = 0.07. However, as would be anticipated for HIV risk transmission, those who usually bled during a clean shave, had a sexually transmitted genital infection or discharge (OR 1.58, p = 0.01) or those with informal piercings or tattoos (OR1.84; p = 0.01), had a greater likelihood of HIV. Even though HCV seroprevalence was low, the trend was similar for HIV/HCV co-infection where likelihood was increased in those who usually bleed after or during a clean shave (OR 2.43; p = 0.02), those with a history of sexually transmitted genital lesions or penile discharge (OR 1.58; p = 0.01) and those with a tattoo/piercing informally done (OR 1.85; p=<0.01). This could be expected as the factors all predict for HIV risk and concomitant HIV positivity, enhances the risk of HCV acquisition.

Finally, in a first study of its kind, we have demonstrated that having *folliculitis keloidalis nuchea*, a dermatological consequence of the unique “chiskop” close shave hairstyle, in of itself does not predict for blood borne infection risk including HIV, HBV and HCV. However associated factors where bleeding is present, may account for a differential in HIV and HIV co-infection with HBV or HCV prevalence. As we have demonstrated, several other variables potentially play a factor as well and thus isolating FKN as a pure risk factor is challenging. Even so, the HIV prevalence in this young male population cohort is concerningly high. HBV rates represent background prevalence of endemic HBV in South Africa, but again prevalence rates are concerningly high. HCV rates are low, and as suggested, may well reflect the lack of involvement of key populations in the study. In conclusion, infection rates are high and underpin the need to ensure enhanced education and access to HIV screening, treatment, and prevention. The challenges of eliminating HIV, HBV and HCV remain, and if anything, the study highlights the urgent need for a comprehensive triple elimination policy in South Africa.

## Study limitations

The COVID pandemic influenced recruitment and paused the study for a period. As noted, key HCV populations were not specifically accounted for in the cohort that may influence rates of HCV, for example. Equally, the lack of a longitudinal follow up precludes clear causality assessment. We also did not assess for severity of FKN (possibly influencing BBI risk) as this fluctuates and at a once off visit, difficult to accurately assess.

## Supporting information

S1 Fig Missing data variables. (PDF)
