# Supplementary material for: Scalp haircuts, keloids and blood-borne virus transmission risk in South Africa—The SHAKA study
Source: PLoS One. 2025 Nov 19;20(11):e0336213. doi: 10.1371/journal.pone.0336213 (PMC12629497; doi:10.1371/journal.pone.0336213)
Supplement: S1 Fig — (PDF) [file pone.0336213.s001.pdf]

Figure 1. Missing data variables

Variable	Number	% (95% CI)
1. Study site	18	1.5 (0.9-2.4)
2. FKN diagnosis	1	0.1 (0.002-0.5)
3. Controls with healthy looking scalp	1	0.1 (0.002-0.5)
4. Consent for HIV, HCV and HBV testing	1	0.1 (0.002-0.5)
5. Born in 1995 and after 1995	3	0.3 (0.1-0.8)
6. Current diagnosis of any scalp ailment	3	0.3 (0.1-0.8)
7. Date of birth	4	0.3 (0.1-0.9)
8. Age at presentation	1	0.1 (0.002-0.5)
9. Study group	1	0.1 (0.002-0.5)
10. Male	1	0.1 (0.002-0.5)
11. Ever had a clean haircut	3	0.3 (0.1-0.8)
12. Frequency of haircuts last month	12	1.0 (0.5-1.8)
13. Use clippers	0	-
14. Razor blade	0	-
15. Shaving cream	0	-
16. Scalp tender/sore after shave	16	1.4 (0.8-2.2)
17. Never bled after a shave	0	-
18. I sometimes bleed	0	-
19. I usually bleed	0	-
20. How many children do you have	7	0.6 (0.2-1.2)
21. Married or co-habiting	7	0.6 (0.2-1.2)
22. Circumcised	4	0.3 (0.1-0.9)
23. If circumcised (method)	2	0.2 (0.02-0.6)
24. Sexual intercourse (penetrative)	3	(0.1-0.8)
25. Regular sexual partners	28	2.4 (1.6-3.5)
26. Sex with person not your regular	21	1.8 (1.1-2.7)
27. Unprotected sex in the past month	22	1.9 (1.2-2.9)
28. Sores or genital discharge before	25	2.2 (1.4-3.2)
29. Ever had anal sex with a male	20	1.7 (1.1-2.6)
30. Ever injected drugs to get high	4	0.3 (0.1-0.9)
31. Smoked drugs/alcohol	6	0.5 (0.2-1.1)
32. Used drugs/alcohol before sex	2	0.2 (0.02-0.6)
33. Vaccinated as a child	12	1.0 (0.5-1.8)
34. Ever received Hep b vaccination	5	0.4 (0.1-1.0)
35. Hep B boosters received	4	0.3 (0.1-0.9)
36. Scarification's from healer	3	0.3 (0.1-0.8)
37. Share a toothbrush with partner	5	0.4 (0.1-1.0)
38. Tattoo/piercing informally done	4	0.3 (0.1-0.9)
